# Total Flavonoids of Rhizoma Drynariae Promotes Differentiation of Osteoblasts and Growth of Bone Graft in Induced Membrane Partly by Activating Wnt/β-Catenin Signaling Pathway

**DOI:** 10.3389/fphar.2021.675470

**Published:** 2021-05-26

**Authors:** Shuyuan Li, Hongliang Zhou, Cheng Hu, Jiabao Yang, Jinfei Ye, Yuexi Zhou, Zige Li, Leilei Chen, Qishi Zhou

**Affiliations:** ^1^ Guangzhou University of Chinese Medicine, Guangzhou, China; ^2^ Lingnan Medical Research Center of Guangzhou University of Chinese Medicine, Guangzhou, China; ^3^ Third Affiliated Hospital, Guangzhou University of Chinese Medicine, Guangzhou, China; ^4^ First Affiliated Hospital, Guangzhou University of Chinese Medicine, Guangzhou, China

**Keywords:** total flavonoids of rhizoma drynariae, induced membrane, Wnt/β-catenin, bone defect, osteogenic efficacy

## Abstract

Total flavonoids of Rhizoma drynariae (TFRD), a Chinese medicine, is widely used in the treatment of fracture, bone defect, osteoporosis and other orthopedic diseases, and has achieved good effects. Purpose of this trial was to explore efficacy of TFRD on bone graft’s mineralization and osteoblasts’ differentiation in Masquelet induced membrane technique in rats. Forty male Sprague-Dawley rats were randomly divided into high dose group (H-TFRD), middle dose group (M-TFRD), low dose group (L-TFRD) and control group (control). The critical size bone defect model of rats was established with 10 rats in each group. Polymethyl methacrylate (PMMA) spacer was implanted into the defect of right femur in rats. After the formation of the induced membrane, autogenous bone was implanted into the induced membrane. After 12 weeks of bone graft, bone tissues in the area of bone graft were examined by X-ray, Micro-CT, hematoxylin-eosin (HE) and Masson trichrome staining to evaluate the growth of the bone graft. The *β*-catenin, c-myc, COL1A1, BMP-2 and OPN in bone graft were quantitatively analyzed by Western blot and Immunohistostaining. Osteoblasts were cultured in the medium containing TFRD. Cell Counting Kit-8 (CCK-8) method, Alkaline phosphatase (ALP) and Alizarin Red S (ARS) staining, Western blot, RT-PCR and other methods were used to detect the effects of TFRD on the proliferation of osteoblasts and the regulation of Wnt/β-catenin signaling pathway. *In vivo* experiments showed that the growth and mineralization of bone graft in TFRD group was better. Moreover, the expression of Wnt/β-catenin and osteogenesis-related proteins in bone tissue of TFRD group was more than that in other groups. *In vitro* experiments indicated that osteoblasts proliferated faster, activity of ALP was higher, number of mineralized nodules and proteins related to osteogenesis were more in TFRD group. But blocking Wnt/β-catenin signaling pathway could limit these effects. Therefore, TFRD could promote mineralization of bone graft and differentiation of osteoblasts in a dose-dependent manner during growing period of the bone graft of induced membrane technique, which is partly related to the activation of Wnt/β-catenin signaling pathway.

## Introduction

Critical-sized defects (CSDs) refers to a bone defect that cannot be healed naturally or treated with a standard cancellous bone graft (Lasanianos et al., 2010). Generally, length of bone loss is more than 2 to 2.5 times the diameter of the affected bone (Wiese and Pape, 2010). It is usually caused by trauma, osteomyelitis and bone tumor resection. Most of the traditional treatments are Papineau bone grafting (Masquelet and Bégué, 2010), vascularized free fibula graft (Osterman and Bora, 1984) and Ilizarov technique (Aronson et al., 1989). Induced membrane technique, also known as masquelet technique, is a new technique for the reconstruction of large bone defects (Masquelet et al., 2000), which mainly includes two steps. In the first step, the bone segments involved by inflammation are thoroughly removed and a fibrous membrane is induced around the bone defect by implanting a Polymethyl methacrylate (PMMA) spacer. In the second step, the induced membrane was opened after 6–8 weeks, the spacer was removed, and autogenous bone was implanted into the induced membrane. Because of its advantages of simple operation, easy fixation and a wide range of indications, it has been continuously concerned by scholars all over the world. Although the successful rate of induced membrane technique is high, it also has the problem of long healing time. Studies have shown that the postoperative bone healing time of induced membrane technique is from 3 to 94 months (Pelissier et al., 2004).

Bone formation is a series of complex physiological and pathological processes, including intramembranous ossification and endochondral ossification. Previous studies have shown that the bone formation function of osteoblasts and the bone resorption function of osteoclasts play a key role in the process of bone formation and remodeling (Raggatt and Partridge, 2010; Chen et al., 2018a). Osteoblasts originate from bone mesenchymal stem cells (BMSCs) and differentiate from BMSCs under the action of osteoblast differentiation factor. Osteoblasts are not only the main effector cells of mechanical stress in bone tissue, but also the main functional cells of bone formation responsible for the synthesis, secretion and mineralization of bone matrix. Its differentiation and proliferation mainly determines the bone mass. Therefore, the regulation of osteoblasts has become an vital target to promote bone formation.

Wnt/β-catenin signaling pathway is an important regulatory mechanism involved in osteoblasts’ differentiation (Krishnan, 2006; Luyten et al., 2009; Milat and Ng, 2009), which is essential for bone development, bone mass maintenance and bone remodeling. When Wnt protein binds to specific frizzled transmembrane receptors and the Low-density lipoprotein receptor related protein (LRP, Lrp5/6) co-receptor on cell surface, β-catenin is released in the cytoplasm and no protein degradation occurs. The accumulated β-catenin is transferred to the nucleus, where it binds to T-cell factor 4 (TCF-4) or lymphoid enhancer factor 1 (LEF-1) to activate downstream target genes such as c-myc, cyclinD, Runx2 transcription (Canalis, 2013). CyclinD 1, β-catenin and c-myc are the main functional molecules in Wnt/β-catenin. Dickkopfs (Dkks) bind and sequester the Lrp5/6 and Krm1/2 membrane complex to inhibit Wnt activity (Monroe et al., 2012). Dkk1 is a secretory Wnt inhibitor with good specificity and is active in many tissues (Pinzone et al., 2009). Data from different animal models have confirmed that Dkk1 can inhibit Wnt signaling pathway and thus inhibit bone formation. Anti-Dkk-1 neutralizing antibodies against the epitopes necessary for LRP-5 and LRP-6 binding to Dkk-1 increased bone mass in normal mice (Glantschnig et al., 2020). The experimental model of multiple myeloma showed that anti-Dkk-1 antibody treatment reversed the inhibitory effect of Dkk-1 on osteoblasts’ differentiation and bone formation, thus reducing bone loss (Fulciniti et al., 2009).

Total flavonoids of Rhizoma Drynariae (TFRD) is an effective ingredient extracted from the dried root of traditional Chinese medicine Rhizoma Drynariae. Nowadays, TFRD has been developed into a postmarketing Chinese medicine called Qianggu capsule (drug approval number: Z20030007, Qi-Huang Pharmaceutical Co. Ltd., Beijing, China) (Sun et al., 2016). TFRD has been wildely used in many Asian countries including China, Korea, and Japan for the treatment of diverse orthopedic diseases such as fracture, osteoporosis, bone defects, arthritis, etc., and has pharmacological activities to promote osteogenesis, anti-inflammation and anti-oxidative damage (Kuo et al., 2014; Song et al., 2016; Yang et al., 2017; Chen et al., 2018b; Jiang et al., 2018). Animal experiments showed that TFRD could increase the number of bone trabeculae and bone mineral density (BMD), improve the morphology of bone tissue, promote new bone formation and increase biomechanical strength in bone defect or osteoporotic rats, and no systemic side effects such as infection were found (Wong et al., 2007; Guo et al., 2019). In addition, *in vitro* experiments showed that TFRD could accelerate the differentiation and mineralization of osteoblasts (Chen et al., 2011) and inhibit the bone resorption of osteoclasts (Jeong et al., 2003). However, its specific mechanism is not clear in terms of microstructure and cellular and molecular biology.

Idea of this trial was to explore effects of TFRD on mineralization of bone graft and osteoblasts’ differentiation in Masquelet induced membrane from the point of view of Wnt/β-catenin signaling pathway. Moreover, it is also hoped to provide experimental basis for TFRD on promoting bone formation and mineralization of induced membrane technique, shortening the treatment cycle of bone healing and improving the quality of osteogenesis.

## Materials and Methods

### Main Materials and Reagents

Total flavonoids of Rhizoma drynariae (TFRD) were purchased from Beijing Qihuang Pharmaceutical Manufacturing Co., Ltd. (National Medicine Permit No. Z20030007, number of production: 04080081, the content of TFRD ≥80%); 4% paraformaldehyde fix solution (Guangzhou Dianzhong Trading Co., Ltd., China, batch number: IS013); Penicillin Sodium for injection (Shandong Lukang Co., Ltd., China, 1.6 million units per bottle, national pharmaceutical standard H37020080); Polymethyl methacrylate (PMMA, Heraeus Company of Germany, batch number: 90914791); Recombinant Human Dickkopf-Related Protein 1/Dkk1(Absin Bioscience Inc., Shanghai, China abs0435); Phosphate-buffered saline (PBS), dulbecco's modified eagle medium (DMEM)/high glucose, trypsin, fetal bovine serum (FBS), Penicillin-Streptomycin, Cell Counting Kit-8 (CCK-8), lipopoly saccharide (LPS), dimethyl lsulfoxide (DMSO) and concanavalin A (ConA) were acquired from Beijing Suo Laibao Technology Co., Ltd. (Beijing, China); Alizarin Red S Staining Quantification Assay, Alkaline phosphatase staining solution, and Alkaline phosphatase Assay Kit were acquired from Guangzhou Haoma Biotechnology Co., Ltd. (Gunagzhou, China).

### Experimental Animals and Groups

Forty healthy male Sprague-Dawley (SD) rats of 10–12 weeks old, weighing 250–310 g (280.3 ± 21.4 g), were selected and provided by Guangdong Medical Experimental Animal Center. License No.: SCXK (Yue) 2018-0002, Experimental Animal Certificate No.44007200064529. All the selected experimental animals were raised in the SPF animal room of Guangzhou University of traditional Chinese Medicine (the laboratory temperature was 22–24°C, the humidity was 60–70%, and the light and dark cycle was 12 h/12 h), feeding feed was provided by the Experimental Animal Center of Guangzhou University of Chinese Medicine. The experiment was carried out one week after feeding. According to the method of random number table, the experimental animals were randomly divided into four groups: high dose group (H-TFRD), middle dose group (M-TFRD), low dose group (L-TFRD) and control group, with 10 rats in each group. All protocols were approved by Institutional Animal Care and Ethics Committee of Guangzhou University of Chinese Medicine.

### The Establishment of Animal Models

**FIGURE 1:**
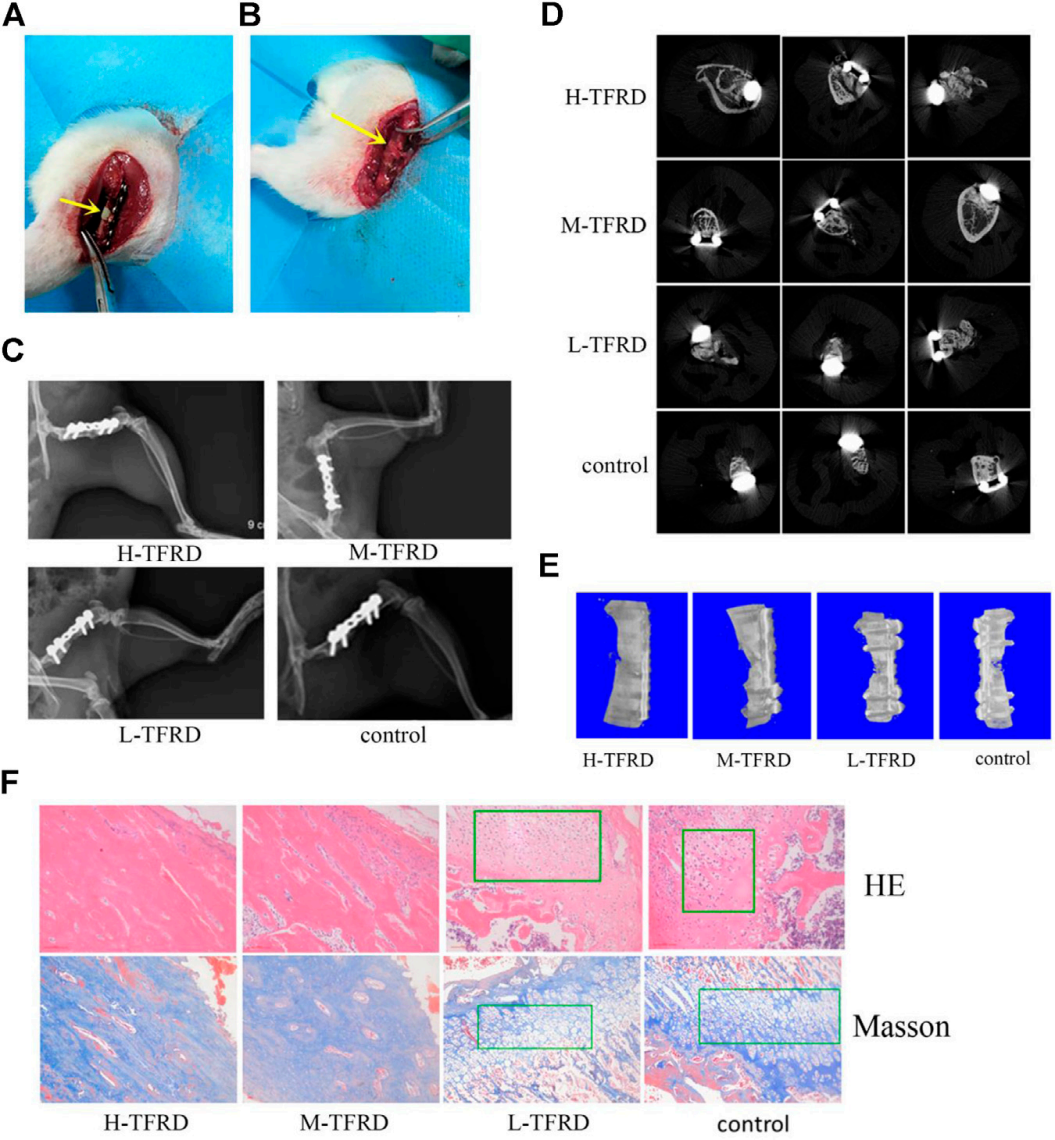
TFRD accelerates the growth and mineralization of bone graft. **(A)** The yellow arrow in the picture refers to the 6 mm bone defect constructed in the right femur of rats during the first stage operation. PMMA spacer was implanted in this area to induce formation of biofilm. **(B)** The yellow arrow refers to the area of bone graft in the right femur of rats at the second stage operation. **(C)** X-ray was performed on the right femur of rats. Among them, the amount of callus and cortical bone shaping in the H-TFRD and M-TFRD groups were more obvious than those in the L-TFRD and control groups. **(D)** was the result of Micro-CT cross-sectional scanning of the bone graft in the right femur of rats. **(E)** was the results of three-dimensional reconstruction of the right femur of rats. **(F)** shows the histological and structural characteristics of bone graft in the right femur of rats (magnification, × 200). The green boxes show the cartilage area, and other parts in pictueres show the osteogenic area.

Surgical procedure was performed as described in earlier work by Gouron et al., with slight modifications (Gouron et al., 2017). Before the experiment, the rats fasted for 12 h, and 40,000 U of penicillin was injected intramuscularly to prevent infection. Anesthesia was given intraperitoneally with 3% pentobarbital (1.5 ml/kg). After the anesthesia took effect, the right hindlimb was shaved to prepare the skin. The rats were taken from the left recumbent position to expose the right hindlimb, sterilized and covered with aseptic towels. The first stage operation: the skin and fascia were cut longitudinally from the lateral greater trochanter to the lateral condyle of the femur, and the subcutaneous muscles were separated to expose the lateral side of the femur. Place a custom six-hole plate on the anterolateral side of the femur. After drilling, two cortical self-tapping screws were used to fix the plate at the distal and proximal ends, and a wire saw was used to cut the bone at the center of the femoral shaft, the length of which was 6 mm. The bone defect area was filled with a PMMA spacer of 6 mm length. After repeated washing with normal saline, the incision was sutured layer by layer (Figure 1A). The second stage operation: six weeks after the first stage operation, two caudal vertebrae were taken from the middle or distal segment of the rat tailbone and cut into particles for bone grafting. The skin, subcutaneous tissue and induced membrane were cut longitudinally along the incision of the first stage operation. After removing the PMMA spacer, the prepared bone particles were filled into the bone defect area (Figure 1B). Finally, the induced membrane, fascia and skin were sutured.

Within 3 days after stage Ⅰ and stage Ⅱ, 40,000 u of penicillin was injected intramuscularly every day to prevent infection.

### Intervention Measures

TFRD was added to distilled water to make a certain concentration of solution. The equivalent dose of TFRD was calculated according to the body surface area. The rats in the high, middle and low dose groups were given TFRD of 0.44, 0.22, and 0.11 g/kg/day respectively, and the rats in the control group were given the same volume of normal saline. In the course of the experiment, the weight was weighed every 2 weeks, and the dose was adjusted in time according to the change of body weight. From the second day after stage II surgery, the rats were administered orally with TFRD until the bone samples were collected after 12 weeks.

### X-Ray, Micro-CT Analyses in Area of Bone Graft

Three rats were taken from each group to analyse by X-ray of femur after 12 weeks of bone graft. The growth and mineralization of bone graft, the bone resorption, loosening or prolapse of steel plates and screws were observed. After X-ray analysis, the rats were killed under excessive anesthesia, the right femur was removed and put into the Micro-CT sample tube for Micro-CT examination. After the completion of the scan, the scanning results were analyzed by CT-An software, and the area of bone graft was manually selected to establish a three-dimensional region of interest and analysis. Micro-CT parameters include Tissue Volume (TV), Bone Volume (BV), Bone Volume Fraction (BV/TV), Bone Surface Fraction (BS/BV), Structural Model Index (SMI), Trabecular Number (Tb.N), Trabecular Thickness (Tb.Th), Trabecular Separation/Spacing (Tb.Sp), Connectivity Density (Conn.D.). After the detection, the three-dimensional reconstruction of the bone graft area of the femur was carried out.

### Histological Analysis

After X-ray and Micro-CT analysis, the bone tissues in the induced membrane was cut and treated with decalcification, dehydration and paraffin embedding, then the tissue slicer was used for continuous slicing with a thickness of 5 μm. After baking at 68°C in a constant temperature baking machine, the bone tissue sections were stained with Masson trichrome and hematoxylin-eosin (HE) staining solution. After sealing, the osteogenic process was observed and evaluated under biological microscope (Olympus, BX53, Japan).

### Immunohistostaining

The paraffin sections of bone tissues were deparaffinized, rehydrated, and then incubated in the citrate antigen retrieval solution (Beijing Solarbio Science and Technology Co.,Ltd., C1031, China) for 5 min. After quenching endogenous peroxidase activity with 3% H_2_O_2_ for 8 min, the slides were incubated with anti-BMP-2 antibody (ab214821, Abcam, United States, 1:100), anti-COL1A1 antibody (ab270993, Abcam, United States, 1:100) and anti-OPN antibody (ab228748, Abcam, United States, 1:100) at 4°C overnight. On the next day, the slides were incubated at 37°C for 20 min with goat anti-rabbit IgG (A32731, Invitrogen, United States, 1:500). After 3,3′-diaminobenzidine (DAB) (Gene Tech Company Ltd., GK5007, China) staining, the slides were counterstained with hematoxylin for 3 min at room temperature, dehydrated in a series of 70–100% alcohol baths and cleared in a xylene bath. The slides were mounted with neutral balsam and observed using a biological microscope (Olympus, BX53, Japan).

### Extraction and Identification of Osteoblasts

Five suckling rats of SD rats were killed, soaked in 75% alcohol for 2 min, the calvaria was taken under strict sterile conditions, the connective tissue attached to the bone surface was removed, PBS was washed repeatedly until the bone tissue was whitened, the bone tissue was cut to the size of 1 mm × 1 mm with scissors, the phosphate buffer (PBS) was rinsed to the bone tissue whitening, and the bone tissue fragments were placed in a centrifuge tube and digested with 0.25% trypsin. After 30 min, trypsin was discarded, 0.1% type II collagenase of 8 ml was added, digested for 1 h, the Supernatant fluid was collected and transferred to another centrifugal tube, and centrifuged by 1000 r/min for 10 min 0.1% type Ⅱ collagenase was added to the centrifuge tube containing bone tissue for 1 h, and the Supernatant fluid was collected and centrifuged to collect cells; added to the prepared cell culture medium. The cells were inoculated in the 25 cm^2^ culture bottle at the concentration of 2 × 10^4 ^/ml and cultured in incubator (37°C, 5% CO_2_). The adhesion and growth of cells were observed every day. After the cells were pasted to the bottom, they were digested and passaged with trypsin. The third generation osteoblasts were used in the experiment. After the cells adhered to the wall, the culture flasks containing primary osteoblasts and passage osteoblasts were observed and photographed under inverted fluorescence microscope. Osteoblasts were identified by morphological observation and ALP staining.

### Preparation and Grouping of Culture Medium

According to the composition of the medium, they were divided into the following four groups: control group, Dkk1 group, TFRD group and TFRD + Dkk1 group. Osteoblasts were cultured in DMEM (high glucose) containing 10% volume of FBS and 1% volume of Penicillin-Streptomycin. Besides, The culture medium of TFRD group contained different doses of TFRD (0, 12.5, 25, 50, 100, and 200 ug/ml, respectively). The concentration of TFRD in the TFRD + Dkk1 group was 100 ug/ml. In the Dkk1 group and TFRD + Dkk1 group, the Recombinant Human Dickkopf-related protein 1 (Dkk1) was added to each medium at a concentration of 0.4 μg/ml. The third generation of osteoblasts were cultured in various pre-prepared media. In the process of osteoblasts culture, the medium was changed every 2 days, and the growing status of the cells was observed.

### Analysis of the Proliferation Rate of Osteoblasts

Proliferation of cells in each group was detected by Cell Counting Kit-8 (CCK-8) method. Osteoblast suspension (100 μl/well) was inoculated in 96-well culture plate and cultured in different media. CCK eight solution was added to each well and incubated in the incubator for 4 h. The absorbance (O.D value) at 450 nm was determined by enzyme labeling instrument.

### Analysis of Alkaline Phosphatase Activity

After 3, 6, 9, and 12 days of cell culture, the activity of ALP of osteoblasts was measured by Para-nitrophenyl phosphate (pNPP). The culture medium was removed, and 0.5% Triton X-100 cell lysate (50 μl) was added at 4°C for 1 h. The 20 μl lysate was taken on a 96-well plate and operated according to the operation table of the Alkaline phosphatase Assay Kit. The OD value was determined by enzyme labeling instrument at 405 nm wavelength. According to the OD value of the sample, the activity value of ALP (U/L) was read on the ALP standard curve.

### Alkaline Phosphatase and Alizarin Red S Staining

After 6 and 21 days of culture, the medium was removed. Osteoblasts were washed with PBS and fixed with 4% paraformaldehyde for 10 min. Using PBS to wash away 4% paraformaldehyde and add ALP staining solution or 1% alizarin red S solution for 20 min. Finally, the amount of ALP or mineralized nodules was observed under the microscope.

### Immunofluorescent Analysis

After the predetermined time of cells culture (3, 6, 9 days), culture medium was quickly absorbed, and dish was washed with cold PBS for 3 times. The cells were fixed with 4% paraformaldehyde for 30 min and washed with PBS for 3 times. Adding 0.25% Triton X100 to plate at 37°C for 10 min and washing plate for 3 times. After drying, adding sealed serum at 37°C for 30 min. Then, cells were briefly washed with PBS and incubated with anti-β-catenin antibody (ab32572, abcam, United States, 1:250) at 4°C overnight. Then, the primary antibody was sucked out and washed by PBS for 3 times. Under the condition of avoiding light, the secondary antibody (goat anti-rabbit IgG, A32731, Invitrogen, United States, 1:500) with FITC labeling was added. After 1 h, the nuclei of cells were stained with DAPI for 10 min. The expression and localization of β-catenin were observed under fluorescence microscope.

### Protein Extraction and Western Blot Analysis

The total proteins of bone tissues and osteoblasts was extracted, and the protein content was measured by BCA assay kit. The proteins were separated by 15% sodium dodecyl sulfate-polyacrylamide gel electrophoresis (SDS-PAGE) and transferred to the polyvinylidene fluoride (PVDF) membrane. After the end of the film transfer, each blot were blocked with 5% skim milk for 1 h. Then the primary antibody was added and incubated at 4°C overnight. After the incubation membrane was washed, the secondary antibody was added. ECL kit was used for photoluminescence development, and GAPDH (ab8245, abcam, United States, 1:1,000) was used as the reference protein. The grayscale values of each band were analyzed by ImageJ software. The information of primary antibodies was listed as follows: β-catenin (ab32572, abcam, United States, 1:5,000), TCF (ab185736, abcam, United States, 1:1,000), LEF-1 (ab137872, abcam, United States, 1:1,000), cyclinD (#2978, Cell Signaling Technology, United States, 1:1,500), c-myc (ab32072, abcam, Unites States, 1:1,000), Runx2 (ab236639, abcam, United States, 1:1,000), COL1A1 (ab270993, abcam, United States, 1:1,000), BMP-2 (ab214821, Abcam, United States, 1:1,000), OPN (ab228748, abcam, United States, 1:1,000).

### RNA Isolation and Real-time PCR

The total RNA in bone tissues and osteoblasts was extracted by Trizol reagent (Invitrogen, Carlsbad, CA, United States) and reverse transcribed into cDNA by PrimeScript RT reagent Kit (Japan, RR037A). Then, the RT-PCR analysis was carried out through using the Prime Script^TM^RT reagent Kit SYBR (Takara, DRR047A). ACTB was used as the reference gene. If the amplification efficiency of target gene and reference gene is close to 100%, 2^-△△Ct^ method was used for RT-PCR analysis. If not, PFAFFL method is more appropriate (Bustin et al., 2009). In this study, the amplification efficiency of all genes was close to 100%, so we used 2^−△△Ct^ method to analyze the relative expression level. The primer sequence of β-catenin, TCF, LEF-1, cyclinD, c-myc, Runx2 and ACTB was list in Table 1.

**TABLE 1:** Prime sequences for RT-PCR.

gene	Primer sequence
β-catenin	F: 5′-AGG​GCA​ATC​CTG​AGG​AAG​AAG​A-3′
	R: 5′-TGC​GTG​AAG​GAC​TGG​GAA​AA-3′
TCF	F: 5′-CGA​GGA​GGT​CAC​ATC​AGT​GG-3′
	R: 5′-AGG​GAC​AGC​ACC​TCA​TCT​GTA-3′
LEF-1	F: 5′-ACA​CGG​ACA​GCG​ACC​TAA​TG-3′
	R: 5′-CTG​CGC​TCT​CCT​TTA​GCG​TA-3′
cyclinD	F: 5′-CCC​ACG​ATT​TCA​TCG​AAC​A-3′
	R: 5′-GGG​TGG​GTT​GGA​AAT​GAA​C-3′
c-myc	F: 5′-ACT​GCG​GTC​TCC​TAA​AGG​TCG-3′
	R: 5′-GAC​CTG​GGG​AAG​CAG​CAA​C-3′
Runx2	F: 5′- CGG​AAT​GCC​TCT​GCT​GTT​AT-3′
	R: 5′- TTC​CCG​AGG​TCC​ATC​TAC​TG-3′
ACTB	F: 5′-TCA​GCA​AGC​AGG​AGT​ACG​ATG-3′
	R: 5′-GTG​TAA​AAC​GCA​GCT​CAG​TAA​CA-3′

### Statistical Analysis

All the data were analyzed by Stata 12.0 software, and the metrological data such as β-catenin, TCF and LEF protein content were expressed as means ± standard deviation (SD). After satisfying the normal distribution, the mean among the four groups were compared by one-way ANOVA, *p* < 0.05 was considered statistically significant.

## Results

### TFRD Accelerates the Growing Rate of Bone Graft

After 12 weeks of bone graft, new bone could be seen in the femoral bone defect area of different doses of TFRD. In the H-TFRD and M-TFRD groups, the continuous callus filled with defects, the volume of callus was larger, and the cortical bone was basically molded. In the L-TFRD and control groups, the volume of callus in the area of bone defect was small, only partially passed through the area of bone defect, and the cortical bone had not been completely molded. The above results showed that TFRD could promote the growth and mineralization of bone graft in the induced membrane, especially in the H-TFRD and M-TFRD groups (Figure 1C).

### TFRD Enhances Mineralization of Bone Graft

The parameters of Micro-CT showed that the Bone Volume Fraction (BV/TV), Trabecular Number (Tb.N), Trabecular Thickness (Tb.Th), Connectivity Density (Conn.D.) in the H-TFRD and M-TFRD groups were significantly higher than those in the L-TFRD and control groups (*p* < 0.05). The Bone Surface Fraction (BS/BV), Structural Model Index (SMI), Trabecular Separation/Spacing (Tb.Sp) were smaller than those in L-TFRD and control groups, and the difference was statistically significant (*p* < 0.05). The above result suggested that TFRD could promote the formation of bone trabeculae of the right femur of rats 12 weeks after bone grafting (Table 2). Images of cross-sectional scan and three-dimensional reconstruction of micro-CT indicated that H-TFRD and M-TFRD groups had more bone mass and better effects of mineralization, and bone defects in L-TFRD and control groups had not been completely healed (Figures 1D,E).

**TABLE 2:** Comparison of the parameters of bone structure in the area of bone graft in each group. Each value was presented as the mean ± SD. *p < 0.05 Vs. the control group; ^#^ *p* < 0.05 Vs. the L-TFRD.

group	n	BV/TV/%	BS/BV/%	SMI	Tb.N/mm^−1^	Tb.Th/mm	Tb.Sp/mm	Conn.D./1/mm^3^
H-TFRD	3	27.85 ± 1.33*^#^	16.33 ± 1.47*^#^	1.53 ± 0.52*^#^	2.63 ± 0.06*^#^	0.25 ± 0.05*^#^	0.25 ± 0.04*^#^	7.18 ± 0.48*^#^
M-TFRD	3	27.40 ± 0.95*^#^	15.80 ± 2.19*^#^	1.47 ± 0.59*^#^	2.64 ± 0.42*^#^	0.28 ± 0.03*^#^	0.27 ± 0.03*^#^	7.29 ± 0.73*^#^
L-TFRD	3	16.00 ± 2.48	21.65 ± 1.71	2.71 ± 0.39	1.59 ± 0.30	0.13 ± 0.02	0.46 ± 0.05	3.33 ± 0.85
Control	3	14.43 ± 1.07	22.50 ± 2.10	2.80 ± 0.39	1.72 ± 0.16	0.12 ± 0.01	0.42 ± 0.03	3.73 ± 0.56

### Histological and Structural Characteristics of Bone Graft

According to the results of HE and Masson trichromatic staining of bone tissues 12 weeks after bone graft in the femur of rats, the cartilage and osteogenic area could be clearly seen in four groups, which showed a typical process of endochondral ossification. Among them, the osteogenic area in H-TFRD and M-TFRD groups were larger than that in L-TFRD and control groups, and the cortical bone was more fully molded. The L-TFRD and control groups were still dominated by cartilage at this stage. The results suggested that the TFRD could promote the process of intrachondral bone formation in the induced membrane, and the formation of bone at H-TFRD and M-TFRD groups was faster and the effect was better (Figure 1F).

### TFRD Promotes Expression of Wnt/β-Catenin and Osteogenesis-Related Proteins in Bone Graft

**FIGURE 2:**
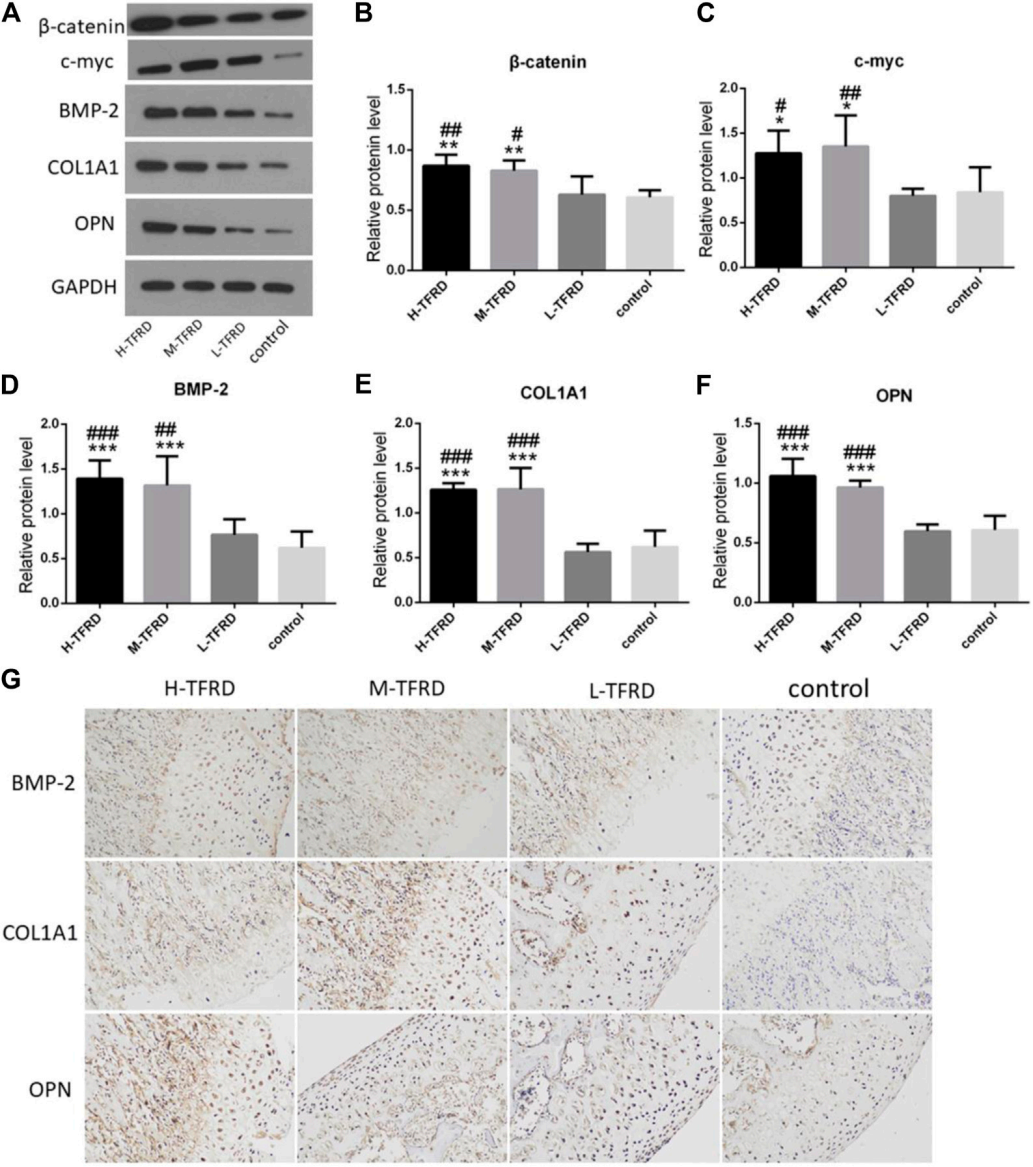
TFRD promotes expression of Wnt/β-catenin and osteogenesis-related proteins in the bone graft. **(A)** is the Western Blot band of β-catenin, c-myc, BMP-2, COL1A1 and OPN respectively. **(B–F)** is the gray value of β-catenin, c-myc, BMP-2, COL1A1 and OPN. Each value was presented as the mean ± SD. ^***^ *p* < 0.001, ^**^ *p* < 0.01, ^*^ *p* < 0.05 vs. the control group; ^###^ *p* < 0.001, ^##^ *p* < 0.01, ^#^ *p* < 0.05 vs. the L-TFRD group. **(G)** Immunohistostaining was performed to evaluate the protein level of BMP-2, COL1A1 and OPN in bone graft area (magnification, × 200). The intensity and area of three osteogenesis-related proteins in the H-TFRD and M-TFRD groups were higher than those in the L-TFRD and control groups.

Wnt/β-catenin signaling pathway plays an crucial role in promoting osteoblasts’ differentiation and bone shape (Krishnan, 2006; Luyten et al., 2009; Milat and Ng, 2009). We found that TFRD could significantly promotes the expression of β-catenin and c-myc in bone tissues in a dose-dependent manner (Figure 2). Furthermore, there are many proteins related to bone formation in the process of osteoblast differentiation, such as bone morphogenetic protein 2 (BMP-2), collagen type I alpha 1 (COL1A1) and osteopontin (OPN). According to results of Western blot of bone tissues, the average protein level of BMP-2, COL1A1 and OPN in the H-TFRD and M-TFRD groups was higher than that in the L-TFRD group and control groups (*p <* 0.001 or *p <* 0.01) (Figure 2). The result of immunohistochemical staining also showed that the positive expression level of BMP-2, COL1A1 and OPN in the H-TFRD and M-TFRD groups were also significantly higher than those in the L-TFRD group and control groups (Figure 2G),which was consistent with the result of Western Blot. Therefore, we speculated that TFRD may promote osteoblasts’ differentiation and expression of osteogenesis-related proteins partly by activating Wnt/β-catenin signaling pathway, then promote bone healing in induced membrane.

### TFRD Increases the Number of Osteoblasts and the Activity of ALP in a Dose-dependent Manner

**FIGURE 3:**
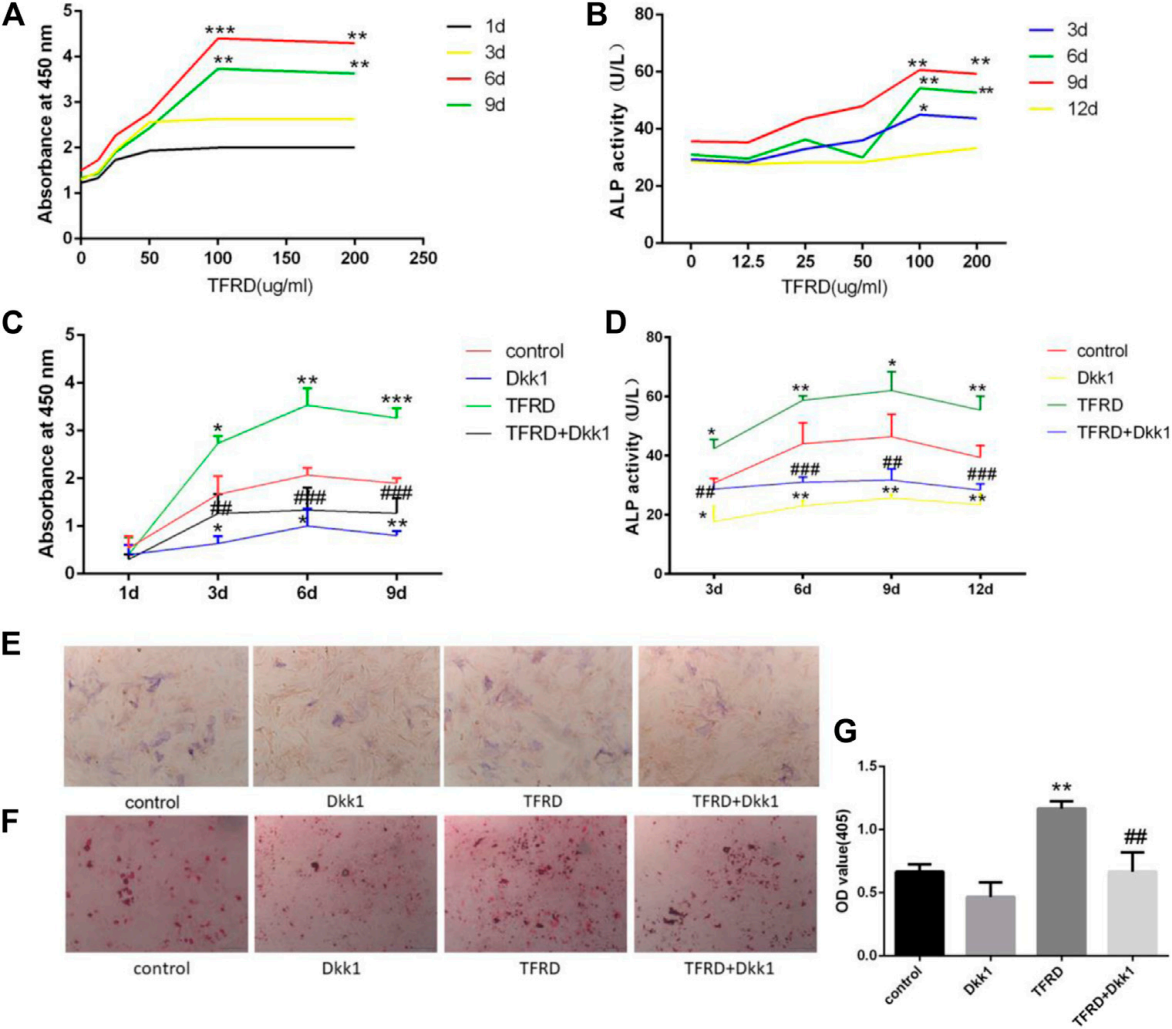
TFRD promotes proliferation and mineralization of osteoblast by Wnt/β-catenin signaling pathway. **(A)** is the effect of different concentrations of TFRD on the proliferation of osteoblasts in different periods. ***p < 0.001, **p < 0.01vs. 0, 12.5, 25, 50 ug/ml. **(B)** is the effect of different concentrations of TFRD on the activity of ALP of osteoblasts in different periods. **p < 0.01, *p < 0.05 vs. 0, 12.5, 25, and 50 ug/ml. (c) is trend and comparison proliferation of osteoblasts in different groups. **(D)** is activity of ALP of osteoblasts in different groups. **(E,F)** The ALP and ARS staining of osteoblasts was performed (magnification, ×100). **(G)** Quantitative determination of ALP activity and the production of mineralized nodules. **(C–G)** N = 3/group. Each value was presented as the mean ± SD. ***p < 0.001, **p < 0.01, *p < 0.05 vs. the control group; ^###^ *p* < 0.001, ^##^ *p* < 0.01 vs. the TFRD group.

In order to determine the effect of the concentration of TFRD on the proliferation of osteoblasts, we used different concentrations of TFRD to culture osteoblasts *in vitro*. The *in vitro* results confirmed that the proliferation rate and activity of ALP from osteoblasts were significantly enhanced in a dose-dependent manner after TFRD treatment, and the proliferative rate and activity of ALP were the highest when the dose of TFRD was 100 μg/ml (Figures 3A,B). Thus, the optimal concentration of TFRD was 100 μg/ml. The follow-up experiments were carried out with the culture medium containing TFRD of 100 μg/ml.

### TFRD Promotes the Proliferation of Osteoblasts by Wnt/β-catenin Signaling Pathway

On the whole, the proliferation of osteoblasts in different groups had a certain rule: during 6 days, osteoblasts’ proliferation increased gradually. The proliferation of osteoblasts entered the plateau on the 6th to 9th day, then decreased. On the 3rd day, 6th day and 9th day, there were significant differences in the increment rate of osteoblasts. The sequence of osteoblasts’ increment rate of each group was as follows: TFRD > control > TFRD + Dkk1 > Dkk1 (Figure 3C). Because Dkk1 is a specific inhibitor of Wnt/β-catenin signalinging pathway, and the increment rate of osteoblasts in TFRD + Dkk1 group was lower than that in TFRD group, the effect that TFRD promoted the proliferation of osteoblasts should be related to the activation of Wnt/β-catenin signaling pathway.

### TFRD Increases the Activity and Amount of ALP of Osteoblasts by Wnt/β-catenin Signaling Pathway

The activity of ALP of osteoblasts in each group also suggested a certain rule: the ALP activity of osteoblasts showed an upward trend during 9 days of cells culture, especially within 3–6 days. From 9 to 12 days, the activity of ALP tended to a downward trend. In our detected time, the activity value of osteoblasts was in the following order: TFRD > control > TFRD + Dkk1 > Dkk1, and the difference was statistically significant (Figure 3D). In order to further analyze the amount of ALP expression in each group, we performed ALP staining after 6 days of osteoblasts culture. The positive region of ALP showed grayish-brown flake deposition in the cytoplasm. The positive region of ALP was the most in the TFRD group, followed by the control group and the TFRD + Dkk1 group, the Dkk1 group was the least (Figure 3E). TFRD could increase the activity and amount of ALP in osteoblasts, but Dkk1 decrease the expression of ALP in osteoblasts.

### TFRD Promotes the Maturation of Osteoblasts

The formation of mineralized nodules is one of the important signs in the process of osteoblasts’ maturation. According to the results of ARS staining of osteoblasts, deep red mineralized nodules were observed in all groups of osteoblasts after 21 days of cell culture, and there were significant differences in the number of mineralized nodules of different groups. Among them, the number of mineralized nodules in the TFRD group was the most, followed by the control and TFRD + Dkk1 group, and the number of mineralized nodules in the Dkk1 group was the least (Figures 3F,G). The results showed that TFRD could promote the maturation of osteoblasts by activating Wnt/β-catenin signaling pathway.

### TFRD Upregulates Wnt/β-catenin Signaling Pathway on Osteoblasts

**FIGURE 4:**
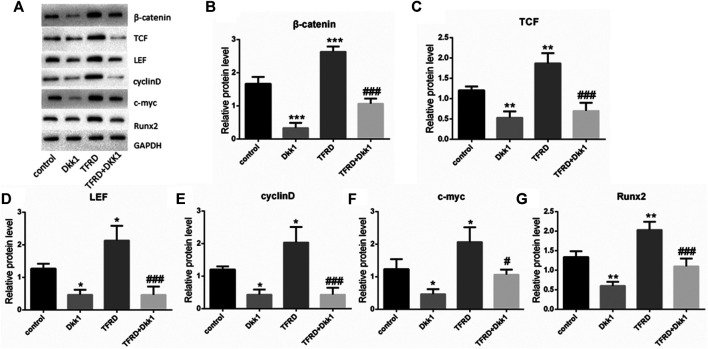
Effect of TFRD on proteins related to Wnt/β-catenin signaling pathway in osteoblasts. **(A)** The relative protein expression levels of β-catenin, TCF, LEF, cyclinD, c-myc and Runx2 were detected by Western blot. **(B–G)** Semi quantitative analysis of protein expression. N = 3/group. Each value was presented as the mean ± SD. ***p < 0.001, **p < 0.01, *p < 0.05 vs. the control group; ^*###*^ *p* < 0.001, ^*#*^ *p* < 0.05 vs. the TFRD group.

**FIGURE 5:**
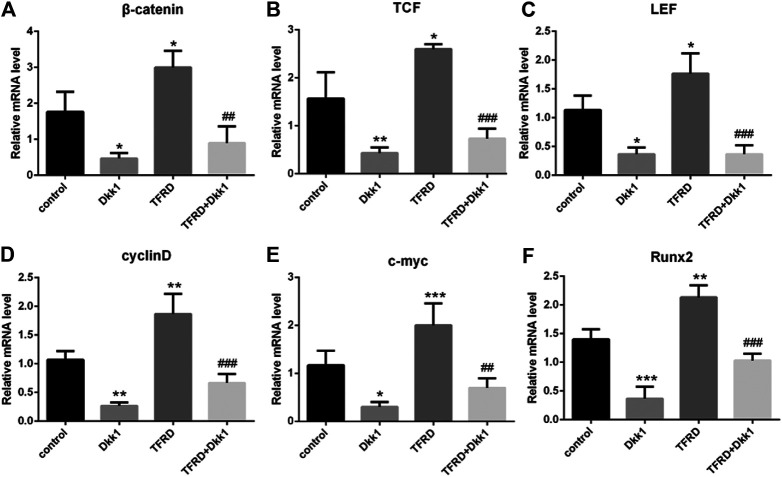
Effect of TFRD on mRNA related to Wnt/β-catenin signaling pathway in osteoblasts. **(A–F)** The relative mRNA levels of β-catenin, TCF, LEF, cyclinD, c-myc and Runx2 were detected by RT-PCR. N = 3/group. Each value was presented as the mean ± SD. ***p < 0.001, **p < 0.01,*p < 0.05 vs. the control group; ^*###*^ *p* < 0.001, ^*##*^ *p* < 0.01 vs. the TFRD group.

According to the results of Western blot, there were significant differences in the expression of β-catenin, TCF, LEF, cyclin D, c-myc and Runx2 of different groups after 6 days of cells culture. The average expression of signaling pathway-related proteins in the TFRD group was higher than that in the control and TFRD + Dkk1 group, indicating that TFRD could promote the activation of Wnt/β-catenin signaling pathway in osteoblasts, but the effect of up-regulation can be contained by Dkk1. At the same time, the control group was higher than the Dkk1 group (Figure 4), demonstrating that Dkk1 had a definite blocking effect on Wnt/β-catenin signaling pathway. In addition, RT-PCR results showed that there were significant differences in the relative mRNA expressions of β-catenin, TCF, LEF, cyclinD, c-myc and Runx2 among different groups. The relative expression of mRNA related to Wnt/β-catenin signaling pathway on osteoblasts was generally consistent with the results of Western blot (Figure 5).

### TFRD Upregulates Wnt/β-Catenin Signaling Pathway during Different Periods

**FIGURE 6:**
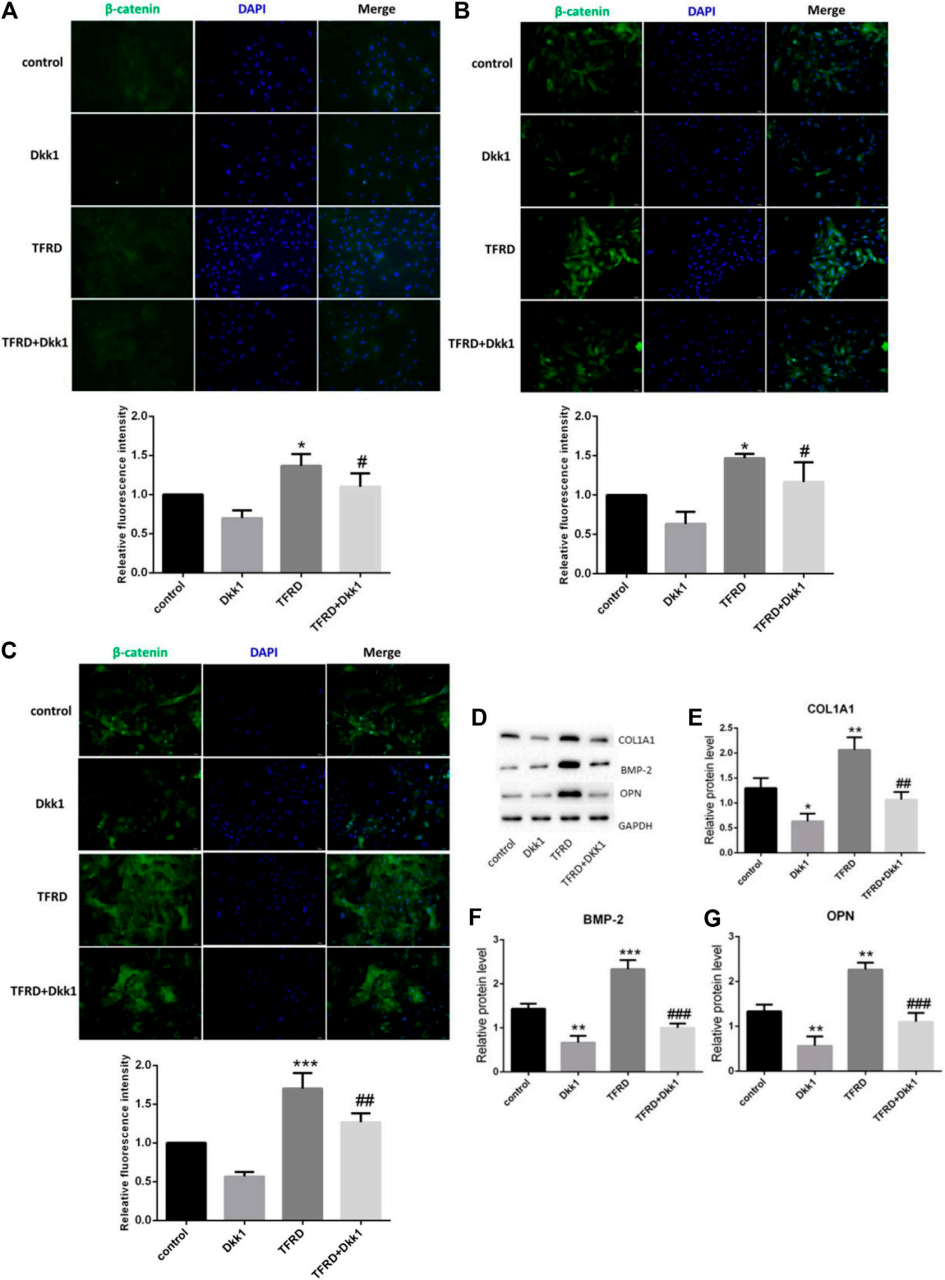
Effect of TFRD on the expression of β-catenin and osteogenic-associated proteins in osteoblasts. **(A–C)** Detection of β-catenin fluorescence in different periods (3, 6, 9 days). The β-catenin was positive in cell membrane, cytoplasm and nucleus, showing high-intensity green fluorescence. Blue fluorescence indicates the nuclei counterstained with DAPI. Values are presented as the mean ± SD of three independent experiments. ***p < 0.001, *p < 0.05 vs. the control group; ^*##*^ *p <* 0.01, ^*#*^ *p* < 0.05 vs. the TFRD group (magnification, × 200). **(D)** The relative protein levels of COL1A1, BMP-2 and OPN were detected by Western blot. **(E–G)** Semi quantitative analysis of protein expression. N = 3/group. Each value was presented as the mean ± SD. ***p < 0.001, **p < 0.01, *p < 0.05 vs. the control group; ^*###*^ *p* < 0.001, ^*##*^ *p <* 0.01 vs. the TFRD group.

In order to dynamically observe the regulatory effect of TFRD on Wnt/β-catenin signaling pathway, the expression of β-catenin protein in different periods was detected by immunofluorescence. The β-catenin was observed in all groups after 3, 6, and 9 days of osteoblasts culture, and was positive in cell membrane, cytoplasm and nucleus, showing high-intensity green fluorescence. The expression region of β-catenin gradually increased with the extension of time. Generally speaking, the fluorescence intensity of β-catenin was more obvious on the 9th day, and was weaker on the 3rd day and 6th day. From the comparison among the groups, the most positive areas of fluorescence signal were found in the TFRD group, followed by the control group. The expression of β-catenin in TFRD + Dkk1 group and Dkk1 group was the least at different stages (Figures 6A–C). These results suggested that TFRD could continuously upregulate Wnt/β-catenin signaling pathway.

### TFRD Promotes Expression of Osteogenesis-Related Proteins

There are many proteins related to bone formation in the process of osteoblast differentiation, such as collagen type I alpha 1 (COL1A1), bone morphogenetic protein 2 (BMP-2) and osteopontin (OPN). We found that TFRD significantly increased the expression of the three osteogenic marker proteins vs. the control group. With the intervention of dkk1, the expression of three osteogenic proteins significantly decreased (Figure 6D). It proved that TFRD may promote the secretion of osteogenesis-related proteins partly by activating Wnt/β-catenin signaling pathway on osteoblasts, then plays the role of osteogenesis.

## Discussion

Although Masquelet technique has achieved a high successful rate in clinical practices, the composition and characteristics of induced membrane and mechanism of its bone healing are not clear. According to the diamond concept of bone healing (Giannoudis et al., 2007; Giannoudis et al., 2008), including osteoblasts, bone conduction scaffolds, blood vessels, osteogenic factors, and the stable mechanical environment, osteoblasts are essential for the growth and mineralization of bone. It is well known that bone formation of osteoblasts and bone resorption of osteoclasts play a key role in the process of bone formation and remodeling. In the process of bone formation, osteoblasts go through four stages: osteoblast proliferation, extracellular matrix maturation, extracellular matrix mineralization and osteoblast apoptosis. Therefore, promoting the proliferation, differentiation and mineralization of osteoblasts and increasing the secretion of osteogenesis-related proteins have become one of the studying idea to accelerate the speed of bone healing in induced membrane.

TFRD promoting bone formation has become a unique method for the treatment of fracture and osteoporosis (Qian, 2015), but the specific mechanism is not clear. An *in vivo* trail indicated that TFRD could increase BMD, mechanical strength, Bone Volume (BV), Bone Volume Fraction (BV/TV), Trabecular Number (Tb.N), Trabecular Thickness (Tb.Th) and decreased Trabecular Separation (Tb.Sp) in osteoporotic rats (Song et al., 2017). Yao *et al.* found that after taking TFRD, chickens with Tibial dyschondroplasia (TD) recovered their walking ability earlier, repair and arrangement of chondrocytes were more regular, the vascular invasion of cartilage area was earlier, and the expression level of BMP-2 and Runx2 were higher (Yao et al., 2018). Both BMP-2 and Runx2 are essential regulatory genes for bone formation and differentiation (Chenard et al., 2012; Nishimura et al., 2012). Most of these trails focus on the effect of TFRD on improving fracture and osteoporosis, but there are few reports on promoting the growth of bone graft after secondary operation of masquelet technique. Our results pointed that remodeling ability of the bone graft in TFRD group was better than that in control group in terms of histology and imaging results.

Studies have shown that bone formation is related to the activation of osteogenesis-related signaling pathways in BMSCs and osteoblasts, including Wnt/β-catenin signaling pathway, MAPK signaling pathway, Smad signaling pathway and so on (Heo et al., 2018). Among these signaling pathways, the most important pathway is Wnt/β-catenin signaling pathway (Huang et al., 2014). With the activation of Wnt/β-catenin signaling pathway on osteoblasts, osteoblasts enter the mitotic phase. At this stage, the differentiation and proliferation of osteoblasts is accelerated, the synthesis of ALP is increased, and calcification is initiated, thus promoting bone formation. The content of ALP in cells represents the degree and state of cell differentiation and is an early specific marker of extracellular matrix maturation (Nguyen et al., 2003). The local use of β-catenin enhancer can promote the proliferation and differentiation of osteoblasts, and then promotes new bone formation (Liu et al., 2010). In a experiment of transgenic mice, it was found that the expression level of β-catenin was directly related to bone formation, and the loss of β-catenin expression directly led to the decrease of osteoblast differentiation and the disturbance of bone formation (Huang et al., 2014). In the Wnt/β-catenin signaling pathway, Dkk1 is one of the vital antagonists, which can specifically inhibit the classical Wnt signaling pathway (Kawano and Kypta, 2003). *In vitro* experiments have confirmed that TFRD can induce the proliferation and differentiation of BMSCs and osteoblasts and inhibit the early apoptosis of osteoblasts (Zhang et al., 2009; Guo et al., 2012). In order to further confirm the specific mechanism of TFRD on osteoblasts, we cultured osteoblasts *in vitro*. The results suggested that TFRD could promote the proliferation and ALP activity of osteoblasts in a dose-dependent manner. After the intervention of the best dose of TFRD (100 ug/ml), the proliferation rate, the activity of ALP and the number of mineralized nodules of osteoblasts in the TFRD group were significantly higher than those in the control group, indicating that TFRD could significantly promote the proliferation and mineralization of osteoblasts. However, this promoting effect of TFRD can be specifically blocked by Dkk1, implicating that the reason that TFRD promotes osteoblasts’ proliferation and mineralization should be related to the up-regulation of Wnt/β-catenin signaling pathway. In addition, the osteoblasts’ increment rate and the expression of pathway protein in the TFRD + Dkk1 group were still higher than those in the Dkk1 group, suggesting that TFRD could reverse the inhibitory effect of Dkk1 on Wnt/β-catenin signaling pathway to some extent.

Moreover, we also explored expression of osteogenic-related proteins, including COL1A1, BMP-2 and OPN, induced by TFRD. COL1A1 is responsible for the synthesis of type 1 collagen, thus ensuring that bones and cartilage are resistant to tension, shear and compression (Palomo et al., 2017). Abnormal collagen production can lead to bone-related diseases, such as Paget disease and osteoporosis (Li et al., 2019). BMP-2 is highly involved in inducing mesenchymal cells to differentiate into osteoblasts and promoting osteoblasts to produce bone matrix (Susperregui et al., 2008). OPN can stimulate osteoblasts’ adhesion, proliferation and calcification, and mediate the changes of bone metabolism caused by mechanical stress (Chatakun et al., 2014). Our experimental results *in vitro* and *in vivo* presented that the expression of these proteins in TFRD group was increased significantly with the increase of TFRD dose. After the intervention of Dkk1, the expression of three proteins decreased, which further confirmed that TFRD promoted the expression of osteogenic-related proteins on osteoblasts by activating Wnt/β-catenin signaling pathway.

Wnt/β-catenin signaling has become an essential pathway for regulating bone formation and bone resorption. However, it is well-known that the aberrant activation of Wnt/β-catenin pathway is the basis of progression of various types of malignant tumors, including colorectal cancer, liver cancer, gastric cancer, lung cancer, breast cancer and so on (Zhao et al., 2018; Zhao et al., 2020; Jiang et al., 2021; Liu et al., 2021; Zheng et al., 2021). TFRD could up-regulate the Wnt/β-catenin signaling pathway, which may potentially aggravate the development of cancers in patients or animals in theory. The failure to verify the safety of TFRD in animals with cancer is one of the limitations of this study. However, we believe that the side effects of medicine, including carcinogenicity, gastrointestinal toxicity, hepatorenal toxicity and so on, are related to population, dose, mode of administration, time and other factors. In the course of this study, two weeks after Masquelet surgery, there were no significant changes in spirit, diet, activity, body weight and skin of these rats. At the end of the experiment, the chest, abdomen, limbs and other parts of the rats did not touch abnormal masses and tumors. In the range of dose and time of our study, TFRD was relatively safe for non-cancer rats. According to the current reports, after TFRD acted on the rat models of bone defect, fracture, osteoporosis and osteonecrosis of femoral head, no carcinogenic and other adverse reactions were found (Song et al., 2016; Song et al., 2017; Yao et al., 2018; Guo et al., 2019; Shen et al., 2020; Lv et al., 2021). The doses of administration in these articles can be summarized as 75–440 mg/kg/d (oral administration) and 20 mg/kg/day (intraperitoneal injection), respectively. Moreover, the mechanism of tumors’ progression is very complex, which is not only one mechanism of aberrant activation of Wnt/β-catenin signaling pathway. TFRD may also up-regulate other protective pathways that inhibit cancers, thus inhibiting the development of cancers. Therefore, whether TFRD has side effects such as aggravating the progression of cancers remains to be determined.

## Conclusion

TFRD could promote the growth and mineralization of bone graft in the induced membrane, which is related to the fact that TFRD should promote osteoblasts’ differentiation, mineralization and expression of osteogenesis-related proteins partly by activating Wnt/β-catenin signaling pathway. However, whether TFRD also upregulates other signaling pathways, whether there is a synergistic effect between these signaling pathways, and side effects of TFRD is the direction of our follow-up studies.

## Data Availability

All relevant data regarding the study is included in this article and any supplementary data is available from the corresponding author upon request.

## Abbreviations

TFRD, Total flavonoids of Rhizome drynariae; TCF, T-cell factor; LEF, lymphoid enhancer factor; HE, Hematoxylin-eosin; Dkk1, Recombinant Human Dickkopf-Related Protein 1; BMSC, bone marrow derived mesenchymal stem cell; DMEM, dulbecco's modified eagle medium; PBS, Phosphate-buffered saline; PMMA, Polymethyl methacrylate; BMD, bone mineral density; ALP, alkaline phosphatase; ARS: Alizarin red S; COL1A1, collagen type I alpha 1; BMP-2, bone morphogenetic protein 2; OPN, osteopontin.
