# Corrigendum: Total Flavonoids of Rhizoma Drynariae Promotes Differentiation of Osteoblasts and Growth of Bone Graft in Induced Membrane Partly by Activating Wnt/β-Catenin Signaling Pathway

**DOI:** 10.3389/fphar.2021.726831

**Published:** 2021-07-16

**Authors:** Shuyuan Li, Hongliang Zhou, Cheng Hu, Jiabao Yang, Jinfei Ye, Yuexi Zhou, Zige Li, Leilei Chen, Qishi Zhou

**Affiliations:** ^1^ Guangzhou University of Chinese Medicine, Guangzhou, China; ^2^ Lingnan Medical Research Center of Guangzhou University of Chinese Medicine, Guangzhou, China; ^3^ Third Affiliated Hospital, Guangzhou University of Chinese Medicine, Guangzhou, China; ^4^ First Affiliated Hospital, Guangzhou University of Chinese Medicine, Guangzhou, China

**Keywords:** total flavonoids of rhizoma drynariae, induced membrane, wnt/β-catenin, bone defect, osteogenic efficacy

In the original article, there was a mistake in Figures 1E, 4A as published. The carelessness in combining the images caused the repetition of the images (Figure 1E H-TFRD and L-TFRD; Figure 4A cyclinD and **Figure 6D** COL1A1).The corrected Figures 1, 4 appear below.

**FIGURE 1:**
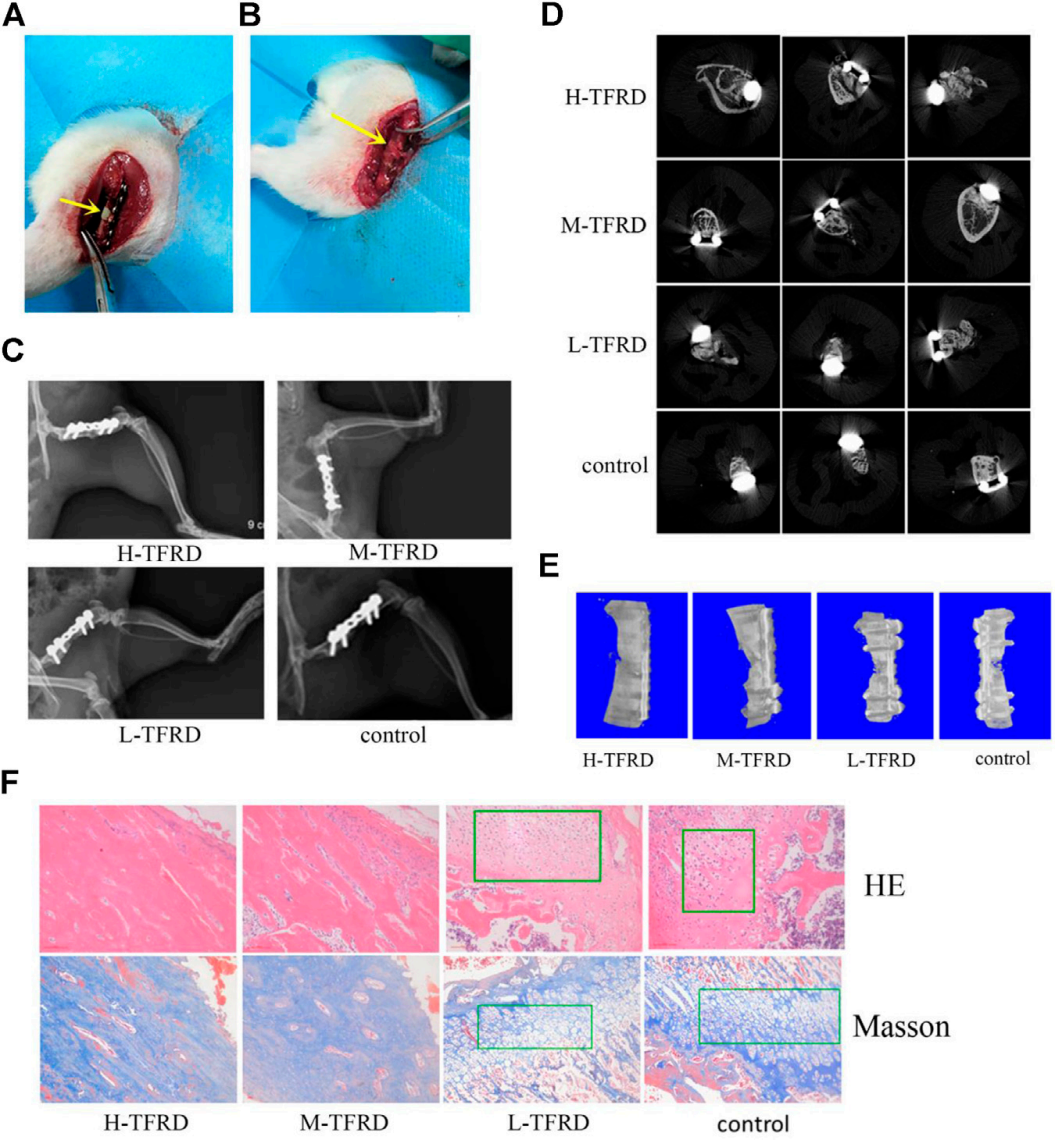
TFRD accelerates the growth and mineralization of bone graft. **(A)** The yellow arrow in the picture refers to the 6 mm bone defect constructed in the right femur of rats during the first stage operation. PMMA spacer was implanted in this area to induce formation of biofilm. **(B)** The yellow arrow refers to the area of bone graft in the right femur of rats at the second stage operation. **(C)** X-ray was performed on the right femur of rats. Among them, the amount of callus and cortical bone shaping in the H-TFRD and M-TFRD groups were more obvious than those in the L-TFRD and control groups. **(D)** was the result of Micro-CT cross-sectional scanning of the bone graft in the right femur of rats. **(E)** was the results of three-dimensional reconstruction of the right femur of rats. **(F)** shows the histological and structural characteristics of bone graft in the right femur of rats (magnification, ×200). The green boxes show the cartilage area, and other parts in pictueres show the osteogenic area.

**FIGURE 4:**
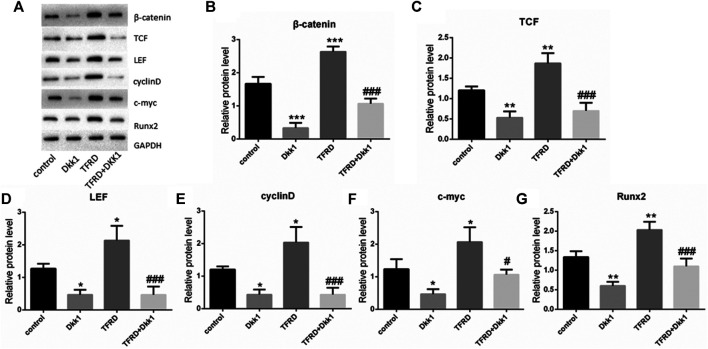
Effect of TFRD on proteins related to Wnt/β-catenin signaling pathway in osteoblasts. **(A)** The relative protein expression levels of β-catenin, TCF, LEF, cyclinD, c-myc, and Runx2 were detected by Western blot. **(B–G)** Semi-quantitative analysis of protein expression. N = 3/group. Each value was presented as the mean ± SD. ***p < 0.001, **p < 0.01, *p < 0.05 vs. the control group; ^###^ *p* < 0.001, ^#^ *p* < 0.05 vs. the TFRD group.

The authors apologize for this error and state that this does not change the scientific conclusions of the article in any way. The original article has been updated.

